# Antiulcer activity of fluvoxamine in rats and its effect on oxidant and antioxidant parameters in stomach tissue

**DOI:** 10.1186/1471-230X-9-36

**Published:** 2009-05-20

**Authors:** Hakan Dursun, Mehmet Bilici, Fatih Albayrak, Cengiz Ozturk, Mustafa B Saglam, Hamit H Alp, Halis Suleyman

**Affiliations:** 1 Department of Internal Medicine, Division of Gastroenterology, Ataturk University, Faculty of Medicine, Erzurum, Turkey; 2 Department of Internal Medicine, Division of Medical Oncology, Ataturk University, Faculty of Medicine, Erzurum, Turkey; 3 Department of Anatomy, Erzurum Numune Hospital, Erzurum, Turkey; 4 Department of Pharmacology, Ataturk University, Faculty of Medicine, Erzurum, Turkey; 5 Department of Biochemistry, Ataturk University, Faculty of Medicine, Erzurum, Turkey

## Abstract

**Background:**

Although many drugs are available for the treatment of gastric ulcers, often these drugs are ineffective. Many antidepressant drugs have been shown to have antiulcer activity in various models of experimental ulcer. One such drug, the antidepressant mirtazapine, has been reported to have an antiulcer effect that involves an increase in antioxidant, and a decrease in oxidant, parameters. To date, however, there is no information available regarding the antiulcer activity for a similar antidepressant, fluvoxamine. This study aimed to investigate the antiulcer effects of fluvoxamine and to determine its relationship with antioxidants.

**Methods:**

Groups of rats fasted for 24 h received fluvoxamine (25, 50, 100 and 200 mg/kg), ranitidine (50 mg/kg) or distilled water by oral gavage. Indomethacin (25 mg/kg) was orally administered to the rats as an ulcerative agent. Six hours after ulcer induction, the stomachs of the rats were excised and an ulcer index determined. Separate groups of rats were treated with the same doses of fluvoxamine and ranitidine, but not with indomethacin, to test effects of these drugs alone on biochemical parameters. The stomachs were evaluated biochemically to determine oxidant and antioxidant parameters. We used one-way ANOVA and least significant difference (LSD) options for data analysis.

**Results:**

The 25, 50, 100 and 200 mg/kg doses of fluvoxamine exerted antiulcer effects of 48.5, 67.5, 82.1 and 96.1%, respectively, compared to the control rat group. Ranitidine showed an 86.5% antiulcer effect. No differences were observed in the absence of indomethacin treatment for any dose of fluvoxamine or for ranitidine. The levels of antioxidant parameters, total glutathione and nitric oxide, were increased in all fluvoxamine groups and in the ranitidine group when compared with the indomethacin-only group. In addition, fluvoxamine and ranitidine decreased the levels of the oxidant parameters, myeloperoxidase and malondialdeyhyde, in the stomach tissues of the rats when compared to indomethacin group.

**Conclusion:**

We conclude that fluvoxamine has antiulcer effects, and that these occur by a mechanism that involves activation of antioxidant parameters and inhibition of some toxic oxidant parameters.

## Background

Steroid and non-steroidal drugs, cigarettes, alcohol usage, trauma, sepsis, shock, *Helicobacter pylori*, and stress have been shown to contribute to gastric ulcer formation [1-4]. Stress is one of the more aggressive factors and underlies many other diseases apart from ulcers, for example, depression. Stress is one of the most commonly used methods to produce ulcer models [5,6]. Depression, accompanied by psychotic and somatic symptoms, is present in most patients with gastro intestinal system (GIS) ulcers [7]. Of interest to the current study are reports that show that certain antidepressants can also have anti-ulcerative effects [8].

The earliest reported use of antidepressants for gastro intestinal (GI) disease was the use of tricyclic antidepressants (TCAs) for the treatment of peptic ulcer disease [8]. Antiulcer effects of some other antidepressant drugs such as fluoksetin, bupropion, dothiepin, maprotiline, mianserin, trimipramine, idazoksan, monoaminooxidase -B (MAO-B) inhibitors, imipramine, amiltriptiline, mirtazapine, among others, have since been reported [9-16]. An increased vulnerability to depression [17] and anxiety [18] in experimental animals is paralleled with ulcer development and the same holds true for humans [19,20]. Moreover, classic antidepressants [21,22] and anxiolytics [23,24] can significantly reduce stress ulcer formation, perhaps to a greater extent than that seen with traditional therapies such as cimetidine and antacids [25].

Fluvoxamine, a selective serotonin reuptake inhibitor (SSRI) drug, inhibits the CYP 1A2 enzyme [26], which is known to produce reactive oxygen species [27]. Nevertheless, etiologic factors are ambiguous in approximately 60%–80% of ulcer diseases, and the physiopathologic conditions in the process of illness are similar [1]. For example, increased levels of reactive oxygen species (ROS) are indicated in the mechanism of both stress and indomethacin-induced gastric damage [28]. The important roles of oxygen-derived ROS and lipid peroxides (LPO) in acute gastric lesions, which are induced by non-steroidal anti-inflammatory drugs (NSAIDs) such as indomethacin, have been supported by experimental data [29,30].

While some SSRI drugs have been reported to enhance upper GIS bleeding when combined with NSAIDs [31-33], fluvoxamine may be beneficial for the GI tract as a consequence of its inhibitory effect on the CYP 1A2 enzyme, and resultant reduction in oxidative damage. The importance of increasing antioxidant parameters and decreasing oxidant parameters in the antiulcer effect mechanism of mirtazapine, an antidepressant drug, has also been reported [16]. To date, however, there is no information available regarding the antiulcer activity of fluvoxamine. The aim of the current study was therefore to examine the effects of fluvoxamine in an indomethacin-induced ulcer model on rats, and to evaluate its effects on oxidant and antioxidant parameters in rat stomach tissue.

## Methods

### Animals

The animals were obtained from the Medical Experimental Research Centre, Atatürk University. A total of 78 male albino Wistar rats, weighing between 190 and 210 g, were used for this study. The animals were fed under normal conditions (22°C) in separate groups. Animal experiments were performed in accordance with national guidelines for the use and care of laboratory animals and were approved by the local animal care committee of Atatürk University.

### Chemicals

All chemicals for laboratory experimentation were purchased from Sigma Chemical (Germany). Indomethacin, fluvoxamine, ranitidine, and thiopental sodium were obtained from Deva Holding-Turkey, Solvay-Turkey, Fako-Turkey and IE Uluagay-Turkey respectively.

### Indomethacin-induced ulcer test

The antiulcer activities of fluvoxamine have been investigated in an indomethacin-induced ulcer models in rats [34]. Fluvoxamine in doses of 25, 50, 100 or 200 mg/kg [35,36] and ranitidine in a 50-mg/kg dose were administered to 24-hour fasted rat groups by oral gavage. An equal volume of distilled water was administered to the control group as a vehicle. Five minutes after drug administration, all groups received 25 mg/kg indomethacin orally. Six hours after indomethacin administration, all rat groups were killed with a high dose of thiopental sodium (50 mg/kg, intra-peritoneal). The stomachs of all of the rats were excised. Ulcer areas on the surfaces of the stomachs were examined macroscopically and measured on square-millimeter paper. The results obtained from the fluvoxamine groups were evaluated by comparing them with those of the control and ranitidine groups. Then, all of the stomachs (fluvoxamine groups, negative control group, positive control group, and intact groups) were kept in -80°C for biochemical investigation of glutathione (GSH), nitric oxide (NO), myeloperoxidase (MPO) and malondialdehyde (MDA) levels.

The groups used for this ulcer experiment can be summarized as follows:

Group 1: Fluvoxamine 25 mg/kg + indomethacin 25 mg/kg

Group 2: Fluvoxamine 50 mg/kg + indomethacin 25 mg/kg

Group 3: Fluvoxamine 100 mg/kg + indomethacin 25 mg/kg

Group 4: Fluvoxamine 200 mg/kg + indomethacin 25 mg/kg

Group 5: Ranitidine 50 mg/kg + indomethacin 25 mg/kg

Group 6: Distilled water+ indomethacin 25 mg/kg

Group 7: Intact group which received only distilled water.

In addition to this ulcer experiment, we evaluated whether administration of fluxoxamine (25, 50 100 and 200 mg/kg doses) and ranitidine (50-mg/kg) to 24 hour fasted rats, in the absence of an indomethacin treatment, would also change biochemical parameters. For this purpose, a total of 36 rats were fasted for 24 hour with free access to water. Then the rats were divided into 6 (n = 6) groups and treated with fluvoxamine at doses of 25, 50, 100 or 200 mg/kg and ranitidine at a 50-mg/kg dose. The last group received only distilled water as vehicle by oral gavage. Six hours after drug administration, all rats in all groups were killed with a high dose of thiopental sodium (50 mg/kg, intra-peritoneal). The stomachs of all of the rats were excised and were evaluated both macroscopically and biochemically, as described above.

Groups for this control experiment can be summarized as follows:

Group 1: Fluvoxamine 25 mg/kg

Group 2: Fluvoxamine 50 mg/kg

Group 3: Fluvoxamine 100 mg/kg

Group 4: Fluvoxamine 200 mg/kg

Group 5: Ranitidine 50 mg/kg

Group 6: Intact group which received only distilled water.

### Biochemical analyses

#### Biochemical investigation of stomach tissues

After the macroscopic analyses, the glutathione (GSH), catalase (CAT), superoxide dismutase (SOD), myeloperoxidase (MPO), and malondialdehyde (MDA) enzyme activities and levels in rat stomach tissues were determined. For this purpose stomachs of rats were frozen at -80°C before until biochemical investigations. To prepare the tissue homogenates, stomach tissues were ground with liquid nitrogen in a mortar. The ground tissues (0.5 g each) were then treated with 4.5 mL of an appropriate buffer. The mixtures were homogenized on ice using an Ultra-Turrax homogenizer for 15 min. Homogenates were filtered and centrifuged using a refrigerated centrifuge at 4°C. The supernatants were used for the determination of the enzymatic activities. All assays were carried out at room temperature in triplicate.

#### Total GSH determination

The amount of GSH in the gastric mucosa was measured according to the method of Sedlak and Lindsay [37]. The mucosal surface of the stomach was collected by scraping, weighed, and then homogenized in 2 mL 50 mM Tris-HCl buffer containing 20 mM EDTA and 0.2 mM sucrose, pH 7.5. The homogenate was immediately precipitated with 0.1 mL of 25% trichloroacetic acid, and the precipitate was removed by centrifugation at 4200 rpm for 40 min at 4°C. The supernatant was used to determine GSH using 5,5'-dithiobis(2-nitrobenzoic acid. Absorbance was measured at 412 nm using a spectrophotometer. The results of the GSH level in the gastric mucosa were expressed as nanomoles per milligram tissue (nmol/mg tissue).

#### NO levels

Tissue NO levels were measured as total nitrite + nitrate levels with the use of the Griess reagent as previously described [38]. The Griess reagent consists of sulfanilamide and N-(1-napthyl)- ethylenediamine. The method is based on a-two-step process. The first step is the conversion of nitrate into nitrite using a nitrate reductase. The second step is the addition of the Griess reagent, which converts nitrite into a deep purple azo compound; photometric measurement of absobance at 540 nm is due to the fact that this azo chromophore accurately determines nitrite concentration. NO levels were expressed as μmol/g.

#### MPO activity

MPO activity was measured according to the modified method of Bradley et al. [39]. The homogenized samples were frozen and thawed three times, and centrifuged at 1500 g for 10 min at 4°C. MPO activity in the supernatant was determined by adding 100 mL of the supernatant to 1.9 mL of 10 mmol/L phosphate buffer (pH 6.0) and 1 mL of 1.5 mmol/L o-dianisidine hydrochloride containing 0.0005% (wt/vol) hydrogen peroxide. The changes in absorbance at 450 nm of each sample were recorded on a UV-vis spectrophotometer. MPO activity in gastric tissues was expressed as minimoles per minute per milligram tissue (mmol/min/mg tissue).

#### Determination of lipid peroxidation or MDA formation

The concentrations of gastric mucosal lipid peroxidation were determined by estimating MDA using the thiobarbituric acid test [40]. Briefly, the rat stomachs were promptly excised and rinsed with cold saline. To minimize the possibility of interference of hemoglobin with free radicals, any blood adhering to the mucosa was carefully removed. The corpus mucosa was scraped, weighed, and homogenized in 10 mL of 100 g/L KCl. The homogenate (0.5 mL) was added to a solution containing 0.2 mL of 80 g/L sodium lauryl sulfate, 1.5 mL of 200 g/L acetic acid, 1.5 mL of 8 g/L 2-thiobarbiturate, and 0.3 mL distilled water. The mixture was incubated at 98°C for 1 h. Upon cooling, 5 mL of n-butanol:pyridine (15:l) was added. The mixture was vortexed for 1 min and centrifuged for 30 min at 4000 rpm. The absorbance of the supernatant was measured at 532 mn. A standard curve was generated using 1,1,3,3-tetramethoxypropane. The recovery was over 90%. The results were expressed as nanomoles MDA per gram wet tissue (nmol/mg tissue).

### Statistical analyses

Data are presented as means ± Standard Error (SE). Enzyme activity and ulcer score results were subjected to one-way ANOVA, with presence of negative and positive controls, using SPSS 11.0 software. Differences among groups were attained using least significant difference (LSD) option, and significance was declared at *P *< 0.05.

## Results

### Indomethacin-induced ulcer test

Macroscopic lesions with evident borderlines in various forms and sizes were dispersed irregularly on all stomach surfaces in the stomach tissue of the control rats which received indomethacin. Hyperemia was more evident in the control group which received indomethacin when compared to the groups which received fluvoxamine and ranitidine. The severity of hyperemia was paralleled with an increase in ulcers. As seen in Table 1, a decrease in ulcer occurrence of 48%, 67.5%, 82.1%, and 96.1% occurred in the stomachs of rat groups which received 25, 50, 100, and 200 mg/kg fluvoxamine. The antiulcer effect of 50 mg/kg ranitidine was 86.5%. No differences were observed in the absence of indomethacin treatment following any dose of fluvoxamine or ranitidine.

**Table 1:** Effects of flovoksamine (FLU) and ranitidine (RAN) on indomethacin (IND) induced ulcers in rats.

Drugs	Dose (mg/kg)	Number of animals	Ulcer area (mm^2^)	Antiulcer effect (%)	P
FLU	25				< 0.05
+		6	15.3 ± 1.5	48.5	
IND	25				

FLU	50				< 0.02
+		6	9.7 ± 0.9	67.5	
IND	25				

FLU	100				< 0.01
+		6	5.3 ± 0.4	82.1	
IND	25				

FLU	200				< 0.001
+		6	1.2 ± 0.02	96.1	
IND	25				

RAN	50				< 0.001
+		6	4.0 ± 0.6	86.5	
IND	25				

Indomethacin (Control)	25	6	29.7 ± 2.6	-	-

### Results of biochemical analyses

**Figure 1:**
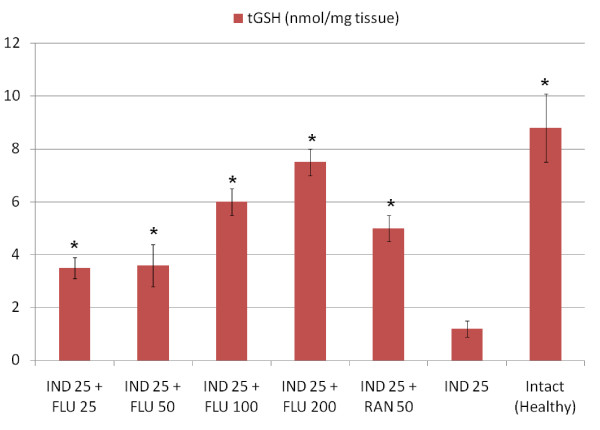
**Effects of fluvoxamine (FLU)+indomethacin (IND), ranitidine (RAN)+indomethacin (IND) and alone indomethacin (IND) on tGSH levels in the stomach tissues of rats**. *Significant at *p *< 0.05 when compared to control.

**Figure 2:**
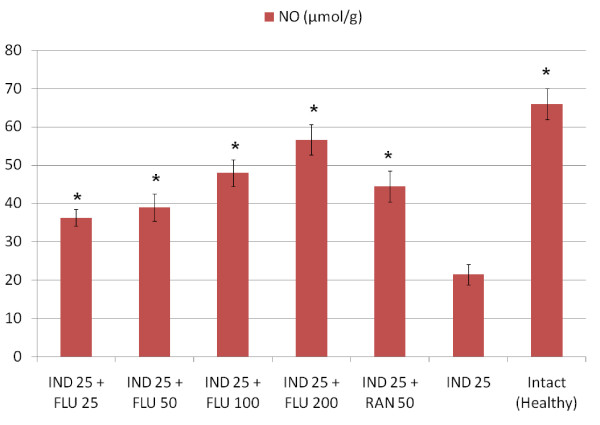
**Effects of fluvoxamine (FLU)+indomethacin (IND), ranitidine (RAN)+indomethacin (IND) and alone indomethacin (IND) on NO levels in the stomach tissues of rats**. *Significant at *p *< 0.05 when compared to control.

**Figure 3:**
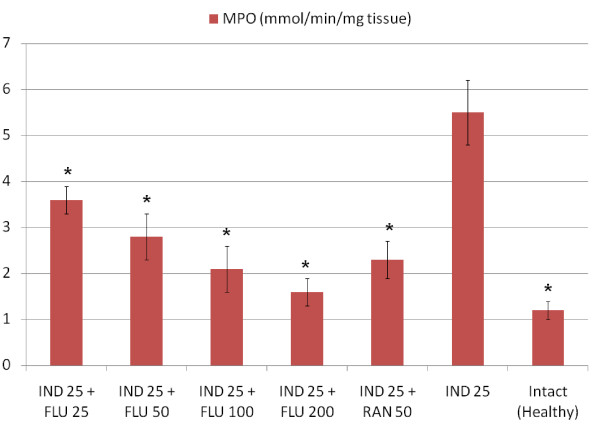
**Effects of fluvoxamine (FLU)+indomethacin (IND), ranitidine (RAN)+indomethacin (IND) and alone indomethacin (IND) on MPO levels in the stomach tissues of rats**. *Significant at *p *< 0.05 when compared to control.

**Figure 4:**
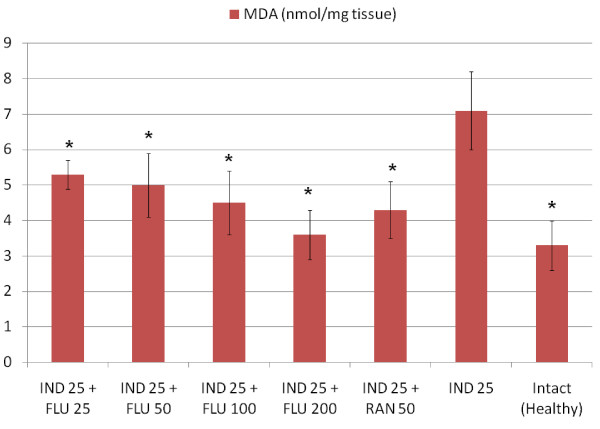
**Effects of fluvoxamine (FLU)+indomethacin (IND), ranitidine (RAN)+indomethacin (IND) and alone indomethacin (IND) on MDA levels in the stomach tissues of rats**. *Significant at *p *< 0.05 when compared to control.

All doses of fluvoxamine and ranitidine significantly decreased the amount of GSH in stomach tissues when compared to healthy intact rats, when administered alone However, a 25 mg/kg dose of fluvoxamine did not affect the levels of other parameters that we measured (MDA, NO and MPO) when compared to healthy intact rats (Table 2). At doses of 50, 100 and 200 mg/kg fluvoxamine significantly decreased the levels of MDA and MPO and increased the level of NO in stomach tissue when compared to intact healthy rat group. Similar changes were observed in tissues from ranitidine-treated rats. Indomethacin application significantly decreased the levels of antioxidant parameters, GSH and NO, and significantly increased the levels of oxidant parameters, MPO and MDA. All doses of fluvoxamine and ranitidine which were co-administrated with indomethacin reversed the negative effects of indomethacin on stomach tissue (Figures 1, 2, 3, 4).

**Table 2:** Effects of fluvoxamine (FLU) alone and ranitidine (RAN) alone on tGSH, NO, MPO, and MDA levels in the stomach tissues of rats.

Drugs	Dose (mg/kg)	tGSH	MDA	NO	MPO
FLU	25	14.9 ± 0.6	2.1 ± 0.3	80.4 ± 6.3	1.0 ± 0.02

FLU	50	15.1 ± 0.3*	1.8 ± 0.2*	95.2 ± 4.5*	0.8 ± 0.04*

FLU	100	17.1 ± 0.8*	1.7 ± 0.3*	99.7 ± 5.7*	0.5 ± 0.03*

FLU	200	18.3 ± 0.6*	1.6 ± 0.2*	105.1 ± 5.2*	0.4 ± 0.01*

RAN	50	15.8 ± 0.4*	2.1 ± 0.5*	96.8 ± 4.7*	0.6 ± 0.03*

Intact (Control)	-	12.7 ± 0.2	2.3 ± 0.4	83.4 ± 3.6	1.1 ± 0.05

*Significant at *p *< 0.05 when compared to intact control.

## Discussion

In this study, the antiulcer effect of fluvoxamine was investigated in rats using an indomethacin-induced ulcer model. In addition, the effect of fluvoxamine on oxidant and antioxidant parameters in rat stomach tissue was evaluated. Fluvoxamine was found to significantly inhibit indomethacin-induced ulcers at all doses tested. The antiulcer capacity of fluvoxamine was determined to be dose-dependent; 200 mg/kg dose of fluvoxamine inhibited indomethacin-induced ulcers more significantly than did ranitidine.

Indomethacin has been shown to produce higher gastric damage in rats when compared to other NSAIDs [41]. For this reason, it has become the preferred drug for inducing ulcer models. In many experimental studies, antidepressant drugs have been shown to produce antiulcer effects by reducing histamine secretion from mast cells, inhibiting gastric acid secretion, and blockading leukotriene (LTC_4_, D_4_, E_4_) receptors [42,43]. Apart from these factors, the important primary factor in indomethacin-induced gastric damage is ROS mediated lipid peroxidation [44] Fluvoxamine, a SSRI drug, inhibits the CYP 1A2 enzyme [26], which is known to produce ROS [27]. For this reason, we investigated fluvoxamine effects on GSH, NO, MPO, and MDA activities in the indomethacin-induced ulcerous stomach tissue of rats, as a first approach at investigating the mechanism behind the antiulcer effects of fluvoxamine.

The roles of toxic oxygen radicals were determined in etiopathogenesis of indomethacin- induced gastric damage [44]. Antioxidant parameters have been shown to be reduced in stomach tissue damaged by indomethacin [45]. Our experimental results are in line with these previous data. Fluvoxamine significantly prevented the negative effect of indomethacin on gastric GSH levels at all doses used. The gastric GSH level was highest at the most effective dose of fluvoxamine. In addition, the GSH level was lower at the 25 mg/kg dose, which had the lowest antiulcer effect. All doses of fluvoxamine and ranitidine also increased the GSH content significantly when administered in the absence of an indomethacin treatment. Our experimental results and the previously published literature data indicate that there is an important relationship between gastric GSH levels and ulcer severity. In tissue, GSH and GSH-related enzymes are accepted as important protective agents due to their antioxidant properties [46].

NSAID treatment causes reduction of these antioxidants and initiates lipid peroxidation in stomach tissue, resulting in gastric damage [47]. All doses of fluvoxamine used in our experiment (25, 50, 100, and 200 mg/kg) restored the GSH levels in gastric tissue which were decreased by indomethacin; this affected the antioxidant defense system positively and reduced gastric damage. GSH detoxifies hydrogen peroxide and/or organic acids chemically; hydrogen peroxide accumulates in the absence of GSH [48]. In the presence of transition metals such as Fe and Cu, hydrogen peroxide reacts with superoxide resulting in the formation of hydroxyl radical, the most reactive and cytotoxic form of ROS [49].

In stomach tissue damaged by indomethacin, NO levels have been shown to be reduced [50]. NO is known to modulate acid levels, gastric mucus secretion, and blood flow in gastric tissues [51]. NO has also been reported to prevent membrane lipid peroxidation [52]. NO levels have been shown to be reduced in damaged stomach tissue [53]. Khattab et al reported that L-arginine almost completely protects against indomethacin-induced gastric ulceration. The mechanism for this is independent of any modulation of acid secretion, mucin content, or pepsin activity, but appears to occur via maintenance of mucosal NO. This study also demonstrated that the NO synthase inhibitor, L-NAME, aggravated ulcer formation [54]. In the current study, all doses of fluvoxamine, which exerted a significant antiulcer effect, also increased gastric NO levels significantly when compared to the control. A parallel between the decrease of NO levels and severity of gastric damage was also noted. Therefore, we can conclude that the antiulcer effects of fluvoxamine could be mitigated by L-NAME application, similar to the effect potentiated by L-arginine application. In our experiment, ranitidine was also shown to increase NO levels significantly when compared to control. Our results are in line with those reported previously in the literature [30].

Indomethacin has been shown to produce damage via increasing mucosal MPO and MDA levels in gastric tissue [55]. MPO exists in polymorph nuclear leukocyte cells (PNL) and catalyses the formation of toxic hypochlorous acid (HOCl) from hydrogen peroxide [56]. In addition, polymorph nuclear leukocytes (PNLs) excessively produce superoxide anion (O2-) and hydroxyl radical (OH-), which are free oxygen radicals [57]. Excessive production of MPO and other reactive radicals cause oxidative damage; oxidative damage is represented by measuring lipid peroxidation levels [58]. Lipid peroxidation is an important reason for cell membrane damage; MDA is the end product of lipid peroxidation and is used to determine lipid peroxidation levels [59]. Gastric MPO and MDA increases resulting from indomethacin application were decreased by fluvoxamine. Fluvoxamine decreased levels of oxidant parameters and increased those of antioxidant parameters not only in rats given indomethacin, but also in healthy, intact rats.

CYP1A2 enzymes are also known to produce ROS [27]. Fluvoxamine inhibited these enzymes [26]. So above mentioned antioxidative effects of fluvoxamine may also be related to inhibition of CYP1A2 enzymes.

Gastric side effects of SSRI drugs have been reported [31]. The combined usage of SSRI drugs and indomethacin has been reported to cause gastrointestinal bleeding [60]. However, several novel arylpiperazine serotonin 1A receptor (5HT1-A) agonists, developed as anxiolytics, were shown to have antisecretory and gastroprotective effects in rats [61]. In addition, 5HT1-A antagonists increase the potency of serotonin-related contractions in stomach tissue [62], while the 5HT1-A agonist buspiron decreases stomach and intestinal distension [63]. In the light of this literature, it can be hypothesized that the antiulcer effect of fluvoxamine may be related to a stimulation of 5HT1-A receptors, but further detailed studies are required to clarify this point.

## Conclusion

In conclusion, we report that fluvoxamine has antiulcer effects. Indomethacin causes gastric damage by not only inhibiting cyto-protective PG synthesis, but also by affecting oxidant and antioxidant mechanisms, such as GSH, NO, MPO, and MDA. Fluvoxamine appears to exert its antiulcer effects by activation of antioxidant mechanisms and inhibition of toxic oxidant mechanisms in stomach tissues.

## Competing interests

None of the authors has a commercial interest, financial interest, and/or other relationship with manufacturers of pharmaceuticals, laboratory supplies, and/or medical devices or with commercial providers of medically related services.

## Authors' contributions

HD participated in the sequence alignment and drafted the manuscript. MB participated in the sequence alignment. FA participated in the design of the study and performed the statistical analysis. CO evaluated the results with an anatomical perspective, and participated in study design. MBS carried out the animal experiments and participated in the sequence alignment. HHA performed the biochemical experiments. HS conceived of the study, and participated in its design and coordination. All authors read and approved the final manuscript.

## Pre-publication history

The pre-publication history for this paper can be accessed here:

http://www.biomedcentral.com/1471-230X/9/36/prepub
